# Drug-Induced Nephrotoxicity and Dose Adjustment Recommendations: Agreement Among Four Drug Information Sources

**DOI:** 10.3390/ijerph120911227

**Published:** 2015-09-09

**Authors:** Millena Drumond Bicalho, Danielly Botelho Soares, Fernando Antonio Botoni, Adriano Max Moreira Reis, Maria Auxiliadora Parreiras Martins

**Affiliations:** 1 Maternidade Hospital Octaviano Neves, Rua Ceará, 186, Bairro Santa Efigênia, Belo Horizonte CEP 30150-310, MG, Brazil; E-Mail: milleninhadb@hotmail.com; 2 Faculdade de Farmácia, Universidade Federal de Minas Gerais, Av. Antônio Carlos, 6627, Campus Pampulha, Belo Horizonte, CEP 31270-901, MG, Brazil; E-Mails: danielly_botelho@yahoo.com.br (D.B.S.); amreis@outlook.com (A.M.M.R.); 3 Faculdade de Medicina, Universidade Federal de Minas Gerais, Av. Alfredo Balena, 190, Belo Horizonte, CEP 30130-100, MG, Brazil; E-Mail: fabotoni@gmail.com; 4 Hospital Risoleta Tolentino Neves, R. das Gabirobas, Bairro Vila Cloris, Belo Horizonte, 31744-012, MG, Brazil

**Keywords:** kidney insufficiency, drug toxicity, drug information

## Abstract

Hospitalized patients require the use of a variety of drugs, many of which individually or in combination have the potential to cause kidney damage. The use of potentially nephrotoxic drugs is often unavoidable, and the need for dose adjustment should be evaluated. This study is aimed at assessing concordance in information on drug-induced nephrotoxicity and dose adjustment recommendations by comparing four drug information sources (DRUGDEX^®^, UpToDate^®^, Medscape^®^ and the Brazilian Therapeutic Formulary) using the formulary of a Brazilian public hospital. A total of 218 drugs were investigated. The global Fleiss’ kappa coefficient was 0.265 for nephrotoxicity (*p <* 0.001; CI 95%, 0.211–0.319) and 0.346 for recommendations (*p <* 0.001; CI 95%, 0.292–0.401), indicating fair concordance among the sources. Anti-infectives and anti-hypertensives were the main drugs cited as nephrotoxic by the different sources. There were no clear definitions for qualitative data or quantitative values for dose adjustments among the four information sources. There was no advice for dosing for a large number of the drugs in the international databases. The National Therapeutic Formulary offered imprecise dose adjustment recommendations for many nephrotoxic drugs. Discrepancies among information sources may have a clinical impact on patient care and contribute to drug-related morbidity and mortality.

## 1. Introduction

Kidney diseases are considered a public health issue worldwide [1,2]. They can become chronic, lead to increased risk of cardiovascular disease [3,4] and raise healthcare costs [5]. In addition, they contribute to approximately 850,000 deaths each year, making them the 12th leading cause of death [2]. The ageing population and advanced life expectancy have contributed to the increased prevalence of chronic diseases, including chronic kidney disease [1]. However, people can develop acute kidney injury (AKI), which is associated with diverse causes, such as burns, shock, drug toxicity, sepsis, trauma and severe diarrhoea, at any age. In the United States, AKI is one of the most serious and common health complications, occurring in up to 20% of all hospitalized patients and in over 45% of patients in a critical care setting [6].

Finlay *et al.* [7] define nephrotoxic drugs (ND) as therapeutic agents that have the potential to cause adverse effects on renal function as a result of direct toxicity or compromised renal perfusion, and this toxicity may depend on the clinical context involved. The types of kidney dysfunction that are induced by ND include acute tubular necrosis, glomerular and tubulointerstitial injury, haemodynamically mediated damage and obstructive nephropathy [8]. Previous studies showed that ND were responsible for 19%–25% of AKI in critically ill patients [9,10,11]. The therapeutic classes cited to be more involved with AKI are antibiotics, non-steroidal anti-inflammatory drugs (NSAIDs), angiotensin-converting enzyme inhibitors (ACE inhibitors) and radiocontrast agents [12,13].

Due to the inherent risks associated with using ND, they should be prescribed with caution and the dosage recommendations should be strictly followed [14]. Access to objective and reliable sources of drug information is critical to supporting appropriate treatment decisions regarding dose adjustments for drugs that affect renal function [15]. Vidal *et al.* [16] noted a disparity in secondary information sources, difficulty in searching for primary information, and recommendations that were elaborated in qualitative and undefined terms with inappropriate adaption for practical use. These factors result in an absence of explicit guidance for how to adjust doses or dosing intervals. Thus, some authors have recommended that health professionals perform a critical analysis of different drug information sources before making decisions [17,18]. This study is aimed at assessing the concordance of information regarding drug-induced nephrotoxicity and dose adjustment recommendations by comparing four drug information sources using the formulary of a Brazilian public hospital.

## 2. Materials and Methods

### 2.1. Design

In this descriptive study, we used four sources of tertiary information as follows: DRUGDEX^®^, accessed 8 Mar 2014 [19]; UpToDate^®^, accessed 24 Mar 2014 [20]; Medscape^®^, accessed 13 Mar 2014 [21] and the last updated version of the National Therapeutic Formulary (NTF), 2010 [22] which is a print publication that is edited by the Brazilian Ministry of Health. DRUGDEX^®^ is a section of the Micromedex database that provides referenced monographs for drugs and is accessed by subscription. UpToDate^®^ is an evidence-based, physician-authored clinical decision support resource that is also available to subscribers. Medscape is a free online reference that provides information on topics related to drug therapy. The selection of these three international databases, which are regularly peer reviewed and updated, was based on their widespread use by healthcare professionals in several countries. NTF was elaborated by Brazilian experts and contains scientific information on the drugs that are listed on the National List of Essential Drugs, a reference that is recommended by the Ministry of Health to guide health practices in the country.

### 2.2. List of Investigated Drugs

The drugs selected for investigation in the information sources were extracted from the drug formulary (updated in 2014) of a public university hospital located in Belo Horizonte in southeast Brazil. The drugs considered in this study are used via oral and parenteral routes of administration. When the same drug was listed for use at different concentrations and in different pharmaceutical forms, it was considered as only one drug. Drugs administered by topical and ophthalmic routes, germicides, inhaled drugs, dialysis solutions and other non-drug products were excluded from the search.

### 2.3. Procedures for Searching on Drug Information Sources

Information on nephrotoxicity and recommendations for dosing were searched for in the computer databases by using the International Nonproprietary Name (INN) for each drug included in this study. A manual review in the NTF was performed to search for drugs by their Brazilian Common Denomination (BCD). The definition of ND that was proposed by Finlay *et al.* [7] was used in the present study. Recommendations for dosing for dialysis and haemodialysis were not compared. The information was extracted from the four sources by one researcher (MDB) who compiled the precise lists into a descriptive table in Microsoft Excel that included the drug name, therapeutic class, information on nephrotoxicity and dose adjustment recommendations, as indicated in the reference. Two authors independently reviewed the content of each reference regarding for information regarding nephrotoxicity (MDB and DBS) and dose adjustment recommendations (MDB and AMMR). Disagreements on the assessment of nephrotoxicity or recommendations for dosing were discussed with a third reviewer (MAPM) until a consensus was reached. A drug was considered to be nephrotoxic when the database mentioned any mechanism that could involve kidney dysfunction or described its risk for causing any degree of renal impairment. Dose adjustment recommendations were separated into six categories according to the categorization described by Khanal *et al.* [15], as follows:

(1) Contraindicated (CI): This category included drugs that were recommended to be avoided in the presence of renal impairment of any severity.
(2) Numerical recommendations (N): Dose modifications were recommended based on the creatinine clearance (CrCl) or eGFR/serum creatinine (SCr) value. Dose modifications based on CrCl/eGFR/SCr were not mentioned, but there was a clear recommendation to avoid the drug below a certain range of CrCl/eGFR/SCr values.
(3) Non-numerical recommendations (NN): Recommendations that were ambiguous when describing the required dose for a particular stage of renal impairment, including severe renal impairment, or that were described without an eGFR/CrCl value/severity of renal impairment for which the drug should be avoided or reduced. This category could also include recommendations to use the medication with caution that were given without specific requirements for dose adjustments based on the CrCl/eGFR/SCr value.
(4) Dosage adjustments not required (NR): The information source advised to give the usual drug dose in the presence of renal impairment.
(5) No advice mentioned (NA): The drug monograph was presented in the information source, but there was no information regarding its use in patients with renal impairment.
(6) Missing (M): This category included drugs that were not found in the information source.

### 2.4. Analysis of Data

The consulted sources were compared in terms of the similarities and inconsistencies that were observed in their information. The frequency of identifying information related to nephrotoxicity and dose adjustment recommendations in each source was determined. To assess agreement among the sources, each of the nephrotoxicity citations was transformed into a binary variable (yes/no) in the database according to its presence or absence in each individual list. Dose adjustment recommendations were described by category and then coded as a binary variable (yes/no) to evaluate their presence (CI, N, NN and NR) or absence (M and NA) in the database. The Fleiss’ kappa coefficient [23] was calculated to evaluate overall concordance, and the Cohen’s kappa coefficient [24] was used to determine the pairwise concordance among the sources and to minimize the influence of the short ND list that was presented by the NTF. DRUGDEX^®^ was considered the standard and was used as a comparison for the other sources. Concordance was evaluated according to the following degrees of agreement for the kappa coefficients proposed by Landis and Koch [25]: <0 = poor, 0.00–0.20 = slight, 0.21–0.40 = fair, 0.41–0.60 = moderate, 0.61–0.80 = substantial, 0.81–0.99 = almost perfect, and 1.00 = perfect). Data were analysed using the Statistical Package for the Social Sciences (SPSS for Windows, version 18.0; SPSS, Chicago, IL, USA).

## 3. Results

**Figure 1:**
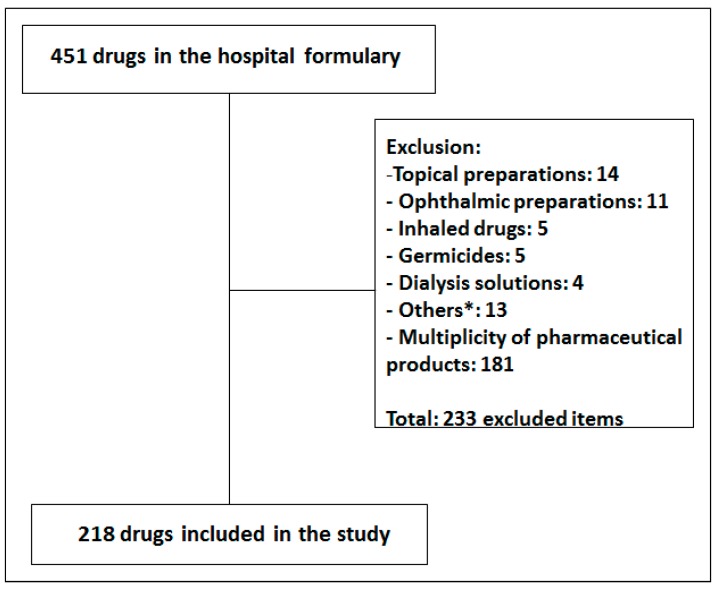
Flow chart for the selection of studied drugs. * Others included: tissue adhesives, diagnostic agents, suppositories, absorbable haemostatic, fibrin sealant, soda lime, activated carbon and absorbable gelatin sponges.

From a total of 451 drugs included in the hospital formulary, 218 were selected to be studied. The details related to drug selection are presented in the Figure 1.

Information on nephrotoxicity and dosage adjustment recommendations exhibited significant heterogeneity among the sources. With respect to the presence of the consulted drugs in the sources, 215 drugs were cited in DRUGDEX^®^, 211 in UpToDate^®^, 198 in Medscape^®^ and 84 in NTF. Specifically, the frequencies of ND that were cited in DRUGDEX^®^, UpToDate^®^, Medscape^®^ and NTF were 61.5% (n = 134), 55.5% (n = 121), 31.7% (n = 69) and 36.7% (n = 80), respectively. Of the 218 drugs, 34 could be retrieved as ND in only one of the international databases (Table 1). Drugs belonging to the radiocontrast agent and non-steroidal anti-inflammatory therapeutic classes were not extensively cited as ND by the information sources searched in this study.

The global Fleiss’ kappa coefficient obtained for nephrotoxicity citations was 0.265 (*p <* 0.001; IC 95%, 0.211–0.319), indicating a fair amount of concordance among the four drug information sources. Regarding the presence of dose adjustment recommendations, DRUGDEX^®^, UpToDate^®^, Medscape^®^ and NTF showed a frequency of 54.6% (n = 119), 61.0% (n = 133), 32.6% (n = 71) and 36.7% (n = 80), respectively. The global Fleiss’ kappa coefficient obtained to assess the presence of recommendations for dosing was 0.346 (*p <* 0.001; IC 95%, 0.292–0.401), indicating a fair amount of concordance among the sources as well.

The pairwise concordance with Cohen´s kappa coefficient showed increased concordance for the presence of nephrotoxicity classification (0.558) and the presence of dosage adjustment recommendations (0.476) for DRUGDEX^®^ *versus* UpToDate^®^, both of which showed a moderate agreement, as shown in Table 2.

**Table 1:** Drugs classified as nephrotoxic in only one of the international databases out of a total of 218 drugs that were evaluated.

DRUGDEX^®^	UpToDate^®^	Medscape^®^
albumin	domperidone	alfentanil
biperiden	doxycycline	chlordiazepoxide
calcium carbonate	haloperidol	
calcium gluconate	heparin	
diltiazem	hydroxyethylstarch	
dobutamine	ivermectine	
epinephrine	mebendazole	
ephedrine	octreotide	
ketamin	pralidoxime	
magnesium sulfate	promethazine	
metaraminol	propranolol	
pyridoxine		
rifampicin and isoniazid		
sodium chloride		
sorbitolstreptokinase		
teicoplanin		
tenoxicam		
thiopental		
tiabendazole		
zidovudine and lamivudine		

**Table 2:** Measures of pairwise concordance of the binary variables for the presence * or absence of citations for nephrotoxicity and dose adjustment recommendations among the four sources of information.

Source Compared to DRUGDEX^®^	Nephrotoxicity	Dose Adjustment Recommendations
UpToDate^®^	0.558	0.476
Medscape^®^	0.315	0.449
National Therapeutic Formulary	0.135	0.382

* Recommendations for dosing that were classified as contraindicated, numerical, non-numerical or dose adjustments not required were considered present in the source.

The categorization of dose adjustment recommendations for the total of 218 drugs examined in the study revealed that the four information sources presented a lack of quantitative recommendations for modifying doses according to CrCl/eGFR/SCr values. DRUGDEX^®^ provided precise recommendations (CI, N and NR) for the highest number of drugs (n = 104), followed by UpToDate^®^ (n = 89). Medscape^®^ showed a high number of listed drugs with no advice (NA) for dose management (n = 127), followed by DRUGDEX^®^ (n = 96) and UpToDate^®^ (n = 78). The NTF failed to provide specific guidance for dosing management and contained a high number of NN recommendations (Table 3).

**Table 3:** Categories of dose adjustment recommendations for the 218 included drugs according to the four sources of information in this study.

Category	Sources			
	DRUGDEX^®^	UpToDate^®^	Medscape^®^	NTF *
Contraindicated (CI)	2	3	-	4
Numerical (N)	64	61	50	42
Non-numerical (NN)	15	44	13	34
Not-required (NR)	38	25	8	-
No advice (NA)	96	78	127	4
Missing	3	7	20	134
Total drugs	218	218	218	218

* National Therapeutic Formulary.

The assessment of information related to the drugs commonly cited as nephrotoxic in the four sources showed that there was significant variation in how they provided advice for dosing (Table 4). For instance, erythromycin and glibenclamide were presented with a different specification for dosing in each source consulted (N, NN, NA, NR). For ceftriaxone, no advice was provided by Medscape^®^, NN dosing was advised by NTF, and dose adjustment was not required by DRUGDEX^®^ or UpToDate^®^. Even for drugs that were listed with precise recommendations (N) in all sources, discrepancies were found in the CrCl cutoff values used to establish adjustments and in the size of dosing modifications and administration intervals. For amikacin, DRUGDEX^®^ recommended 12–15 mg/kg/dose 2–3 times per week for ClCr < 30 mL/min; Medscape^®^ provided no advice for dosing but recommended intervals of 48 hours between doses when ClCr = 10–25 mL/min, UpToDate^®^ advised the administration once per 24 hours for ClCr = 20–40 mL/min, and NTF recommended administering 30%–70% of the usual dose every 12 to 18 hours when ClCr = 10–50 mL/min.

In addition to heterogeneity in the categorization of recommendations, our examination of individual sources also revealed contradictory information (Table 5). When one source described a drug that required a dose modification in patients with renal impairment, this requirement was often missing from other sources, and in some sources, there would be no advice at all. With regard to neostigmine, for example, guidance varied from no advice in Medscape^®^, no clear specification in NTF (which advised “adjust dose in renal impairment”), a precise dose adjustment in UpToDate^®^, and no adjustment required in DRUGDEX^®^.

## 4. Discussion

The results showed low agreement among the sources for drug-induced nephrotoxicity (kappa = 0.265) and dose adjustment recommendations (kappa = 0.346). Some medications cited as nephrotoxic were not even listed in some of the studied databases. Previous evidence was mainly focused on comparing recommendations for dosing across different sources. Khanal *et al.* [15] demonstrated poor agreement (kappa = 0.30) among five sources of information regarding recommendations for use of drugs associated with a risk of renal dysfunction. This study showed a lack of definitions in the qualitative data and a lack of consistency in the quantitative values used for dose adjustments. Product information for different brands of the same drug lacked a standardization in the recommendations for their dose management [26]. In an analysis of 100 drugs, Vidal *et al.* [16] found significant variation in the definitions and recommendations provided for dose adjustments in patients with renal impairment across four sources of information. The evidence for those recommendations was not detailed by the studied sources. These findings are consistent with the results found in the present study.

**Table 4:** Categories of dose adjustment recommendations for drugs commonly cited as nephrotoxic by the four information sources (n = 28).

Therapeutical Class	Drug	Sources			
		DRUGDE^®^	UpToDate^®^	Medscape^®^	NTF *
Anti-infectives	amikacin	N	N	N	N
	amoxicillin	N	N	N	N
	amphotericin B	N	N	N	N
	benzylpenicillin	N	N	N	N
	cefotaxime	N	N	NA	N
	ceftazidime	N	N	N	N
	ceftriaxone	NR	NR	NA	NN
	ciprofloxacin	N	N	N	N
	clarithromycin	N	N	N	NN
	erythromycin	N	NA	NR	NN
	gentamicin	N	N	N	N
	metronidazole	N	N	NA	N
	nitrofurantoin	N	NN	NN	N
	oxacillin	NR	NA	NA	NN
	pyrazinamide	N	N	NA	N
	sulfadiazine	NN	NA	NA	NN
	vancomycin	N	N	N	NN
Anti-hypertensives	acetazolamide	N	N	N	N
	captopril	NN	N	NA	N
	furosemide	N	N	N	NN
	hydralazine	N	N	NA	N
	hydrochlorothiazide	N	NN	N	CI
	losartan	NR	NR	NA	N
	mannitol	CI	NN	NA	CI
Analgesics and antipyretics	paracetamol	N	N	N	N
Anticoagulants	warfarin	NR	NR	NA	NN
Antipsychotics	risperidone	N	N	N	NN
Hypoglycemic	glibenclamide	NR	NA	N	NN

**Table 5:** Examples of discrepancies in recommendations across the information sources.

Drugs	DRUGDEX^®^	UpToDate^®^	Medscape^®^	NTF
amphotericin B	ClCr > 10 mL/min: no adjustment required. ClCr < 10 mL/min: 20–50 mg every 24–36 hours.	If renal dysfunction is drug-induced, the total daily dose can be reduced in 50% or given in alternate days.	ClCr < 10 mL/min: 0.5–0.7 mg/kg IV every 24–48 hours. Consider other antifungal agents with less nephrotoxicity.	ClCr 10–50 mL/min: doses every 24 hours. ClCr < 10 mL/min: doses every 24 or 36 hours.
bumetanide	No adjustment required.	Use is contraindicated in anuria. Use with caution in renal impairment.	No advice.	Missing.
carbamazepine	No adjustment required.	ClCr < 10 mL/min: administrate 75% of dose.	ClCr < 10 mL/min: administrate 75% of dose and monitor.	Missing.
fluoxetine	No adjustment required.	Use with caution. Drug accumulation may occur.	Use with caution. Drug accumulation may occur.	Missing.
heparin	No adjustment required.	No dose adjustment provided in the label of the manufacturer.	No advice.	Severe renal impairment: contraindicated. Renal impairment: reduce dose due to increased risk of bleeding.
losartan	No adjustment required.	No adjustment required.	No advice.	ClCr < 20 mL/min: reduce dose. Recommended: 25mg.
methylene blue	Dose adjustment should be considered (specific recommendations unavailable)	Be cautious in case of severe renal impairment.	No adjustment required. Be cautious in case of severe renal impairment.	Missing.
neostigmine	No adjustment required.	ClCr > 50 ml/min: no adjustment required. ClCr 10–50 mL/min: administrate 50% of normal dose. ClCr < 10 mL/min: administrate 25% of normal dose.	No advice.	Adjust dose in renal impairment.
rifampicin	ClCr ≤ 50 mL/min: 50%–100% of total dose.	No adjustment required.	No advice.	ClCr ≥ 50 mL/min: 100% of dose. ClCr < 49 mL/min: 50% of recommended dose. No need to adjust the dose if it does not exceed 600 mg/day and liver function is normal.
verapamil	No adjustment required.	Use with caution.	Use with caution. Manufacturer recommends 100 mg at bedtime. ClCr < 10 mL/min: reduce the dose in 25%–50%.	Missing.

Disagreement among different sources may extend to other aspects of pharmacotherapy. Comparing listed drug interactions among compendia, Abarca *et al.* [17] noted substantial disagreement among four sources regarding high-risk drug interactions for medications commonly prescribed in the community. Another study demonstrated five individual interactions that were rarely listed in more than one compendium [27]. Martins *et al.* [18] specifically analysed citations used for warfarin interactions and demonstrated that there was poor agreement among five sources (kappa = −0.0080). Other authors have investigated agreement among the top five US drug compendia in terms of drug-induced hepatotoxicity and risk ratings. Their results showed significant discrepancies in the number of hepatotoxic drugs and the severity of hepatotoxicity cited in the databases [28], which confirms a lack of overall consistency observed among different drug information sources.

The heterogeneity of evidence provided by these sources to health professionals may result in non-uniformity in decision making related to patient care. The choice of a source with scarce or non-specific information regarding drug-induced nephrotoxicity may discourage the professional from performing a new search. Neglecting clinically relevant information may directly affect the patient and contribute to the occurrence or progression of kidney damage. Salgado *et al.* [29,30] highlighted the fact that even the current versions of the Summaries of Product Characteristics (SmPCs) that was approved by the European Medicines Agency (EMA) contains several information deficits and recommendations that are not relevant to clinical practice in terms of dose adjustments for patients with renal impairment.

In our assessment of therapeutic classes, anti-infectives and anti-hypertensive drugs, including ACE inhibitors, were the predominant as ND among the sources. These results are consistent with another study that demonstrated that antibiotics, NSAID and ACE inhibitors contain drugs with risk of inducing kidney damage [12]. The drugs listed in the hospital formulary had a small number of NSAID and radiocontrast agents, which led to a limited evaluation of these therapeutic classes among the compendia in this study. However, their potential to induce nephrotoxicity deserves to be highlighted. The mechanism proposed for NSAID involves renal damage that is related to the reduced effect of vasodilator prostaglandins, which causes renal vasoconstriction, a decreased glomerular filtration rate and progression to acute tubular necrosis [31]. The pathophysiology of contrast-associated nephrotoxicity is not well understood. It appears to be a result of direct contrast-induced renal tubular epithelial cell toxicity and renal medullary ischaemia. Furthermore, a key mechanism for this action seems to be the alteration of renal dynamics, which is probably caused by imbalances between vasodilating and vasoconstricting factors [32].

The high potential of anti-bacterials to induce nephrotoxicity has been emphasized by several authors. The proposed mechanism of toxicity is direct injury to the tubular cells [33,34], resulting in acute tubular necrosis, osmotic nephrosis, and crystalline nephropathy [35], in addition to damage to interstitial cells, which causes acute interstitial nephritis [33,36]. Apart from the potential of a drug to induce nephrotoxicity, it is important to consider its pharmacokinetics before planning interventions. For instance, when opiates are used for pain relief in patients with end-stage chronic kidney disease, these drugs can accumulate in the kidneys and subsequently require a dose reduction to avoid local damage. In addition, their active metabolites accumulate in patients with renal failure, which leads to an increased risk of narcosis [37].

Individual analysis of the studied information sources in this study showed that DRUGDEX^®^ and UpToDate^®^ were more user-friendly and enabled the retrieval of additional references, but access to these databases depends on a subscription, and a high number of drugs were cited with no advice regarding the need to manage the dose or any method to do so that was based on renal function. UpToDate^®^ often referred to the absence of information on the package insert provided by the product manufacturer, suggesting deficits in the use of a reference that is frequently incomplete [30]. In contrast, the Medscape^®^ is a free access database, but it also presented expressive limitations in terms of the number of drugs listed, the lack of information on the potential for drug nephrotoxicity and recommendations for dose management in patients with renal impairment. Finally, most drugs were missing in NTF because it is based on the restricted national list of essential drugs, which is focused on primary care and hospitals covering clinical conditions with low complexity. However, as a reference that is elaborated by specialists, more precise information was expected, considering the similar number of numerical (41) and non-numerical (32) recommendations for dose adjustments.

The variety of renal disorders mentioned in the sources studied for this report did not always reflect clinical complications. Thus, drug classifications for nephrotoxicity introduced some difficulties into our interpretations because the criteria for deeming a renal alteration to be mild or severe depended on the concept of nephrotoxicity that was adopted in the source. This approach may have resulted in an overestimation of the total number of ND. The NTF alone may have underestimated the number of potential ND and the spectrum of dosing recommendations due to the limited number of drugs included in the national essential list. In addition, the information was not clear on the specific ability of a drug to induce acute or chronic effects. Probably, these effects could be altered by the clinical status and other drugs used in combination. For drugs that are eliminated as essentially unchanged by the kidneys, the risk of dose-dependent adverse drug reactions increases with decreasing renal function [38] and can be reduced by dose adjustment. It is worth noting that dosage adjustment recommendations can involve either a reduction in the dose or an expanded dose interval for ND and drugs with exclusive renal excretion.

The clinical impact that results from disparities in information that are related to the management of the dose of drugs in patients with renal impairment may be relevant in patient care, especially across hospitals, and may contribute to drug-related morbidity and mortality. Improvements in access to tertiary databases are important to providing decision support tools that are based on scientific evidence, and they must be periodically updated and coordinated by an editorial board. Changes are needed in the materials provided by the manufacturers to better support clinical practice. Even when renal failure had no impact on dosing, this information should be explicit in the database to differentiate, whether no dose adaptation is necessary or no evidence is available to support the recommendation [39]. Hospitals should also invest in effective multidisciplinary teams that include pharmacists to help improve patient safety.

## 5. Conclusions

An analysis of four drug information sources showed that there is significant heterogeneity in the definition of ND and in dosage adjustment recommendations for patients with kidney dysfunction. Anti-infectives and anti-hypertensives were the main therapeutic classes cited as nephrotoxic by the four information sources. There were no clear definitions for qualitative data or quantitative values for dose adjustments. A large number of the drugs were presented with no advice for dosing in the international databases, and NTF offered imprecise dose adjustments for a large number of ND. Discrepancies among information sources may have a clinical impact on patient care and contribute to drug-related morbidity and mortality. Additional studies are needed to assess the quality of drug information sources related to dosing management in patients with decreased renal function.

## References

[1] Nasri H. (2014). World kidney day. Chronic kidney disease and aging: A global health alert. Iran. J. Public Health.

[2] Schieppati A., Remuzzi G. (2005). Chronic renal diseases as a public health problem: Epidemiology, social, and economic implications. Kidney Int. Suppl..

[3] Vlagopoulos P.T., Sarnak M.J. (2005). Traditional and nontraditional cardiovascular risk factors in chronic kidney disease. Med. Clin. N. Am..

[4] Marcen R. (2006). Cardiovascular risk factors in renal transplantation—Current controversies. Nephrol. Dial. Transplant..

[5] McLaughlin K., Manns B., Culleton B., Donaldson C., Taub K. (2001). An economic evaluation of early *versus* late referral of patients with progressive renal insufficiency. Am. J. Kidney Dis..

[6] National Kidney Foundation (NKF) (2015). Acute Kidney Injury (AKI). https://www.kidney.org/atoz/content/AcuteKidneyInjury.

[7] Finlay S., Bray B., Lewington A.J., Hunter-Rowe C.T., Banerjee A., Atkinson J.M., Jones M.C. (2013). Identification of risk factors associated with acute kidney injury in patients admitted to acute medical units. Clin. Med..

[8] Taber S.S., Mueller B.A. (2006). Drug-associated renal dysfunction. Crit. Care Clin..

[9] Mehta R.L., Pascual M.T., Soroko S., Savage B.R., Himmelfarb J., Ikizler T.A., Paganini E.P., Chertow G.M. (2004). Spectrum of acute renal failure in the intensive care unit: The PICARD experience. Kidney Int..

[10] Uchino S., Kellum J.A., Bellomo R., Doig G.S., Morimatsu H., Morgera S., Schetz M., Tan I., Bouman C., Macedo E. (2005). Acute renal failure in critically ill patients: A multinational, multicenter study. JAMA.

[11] Naughton C.A. (2008). Drug-induced nephrotoxicity. Am. Fam. Phys..

[12] Singh N.P., Ganguli A., Prakash A. (2003). Drug-induced kidney diseases. J. Assoc. Phys. India.

[13] Nolin T.D., Himmelfarb J. (2010). Mechanisms of drug-induced nephrotoxicity. Handb. Exp. Pharmacol..

[14] Guo X., Nzerue C. (2002). How to prevent, recognize, and treat drug-induced nephrotoxicity. Clevel. Clin. J. Med..

[15] Khanal A., Castelino R.L., Peterson G.M., Jose M.D. (2014). Dose adjustment guidelines for medications in patients with renal impairment: How consistent are drug information sources?. Intern. Med. J..

[16] Vidal L., Shavit M., Fraser A., Paul M., Leibovici L. (2005). Systematic comparison of four sources of drug information regarding adjustment of dose for renal function. BMJ.

[17] Abarca J., Malone D.C., Armstrong E.P., Grizzle A.J., Hansten P.D., van Bergen R.C., Lipton R.B. (2004). Concordance of severity ratings provided in four drug interaction compendia. J. Am. Pharm. Assoc..

[18] Martins M.A.P., Carlos P.P.S., Ribeiro D.D., Nobre V.A., Cesar C.C., Rocha M.O.C., Ribeiro A.L.P. (2011). Warfarin drug interactions: A comparative evaluation of the lists provided by five information sources. Eur. J. Clin. Pharmacol..

[19] Micromedex (2014). DRUG-REAX^®^ System (Electronic Version).

[20] UpToDate. http://www.UpToDate.com/home/index.html.

[21] MEDSCAPE (Internet) (2014). Copyright^©^ 1994–2014. http//reference.medscape.com/pharmacists.

[22] (2010). National Therapeutic Formulary. Formulário Terapêutico Nacional–FTN. 2010. Rename.

[23] Fleiss J.L. (1971). Measuring nominal scale agreement among many raters. Psychol. Bull..

[24] Cohen J. (1968). Weighted kappa: Nominal scale agreement with provision for scaled disagreement or partial credit. Psychol. Bull..

[25] Landis J.R., Koch G.G. (1977). An application of hierarchical kappa-type statistics in the assessment of majority agreement among multiple observers. Biometrics.

[26] Khanal A., Peterson G.M., Castelino R.L., Jose M.D. (2014). Renal drug dosing recommendations: Evaluation of product information for brands of the same drug. Intern. Med. J..

[27] Fulda T.R., Valuck R.J., Zanden J.V., Parker S., Byrns P.J. (2000). Disagreement among drug compendia on inclusion and ratings of drug-drug interactions. Curr. Ther. Res..

[28] Guo J.J., Wigle P.R., Lammers K., Vu O. (2005). Comparison of potentially hepatotoxic drugs among major US drug compendia. Res. Soc. Adm. Pharm..

[29] Salgado T.M., Arguello B., Martinez-Martinez F., Benrimoj S.I., Fernandez-Llimos F. (2013). Clinical relevance of information in the Summaries of Product Characteristics for dose adjustment in renal impairment. Eur. J. Clin. Pharmacol..

[30] Salgado T.M., Arguello B., Martinez-Martinez F., Benrimoj S.I., Fernandez-Llimos F. (2015). Lack of harmonisation in the classification of renal impairment in European Summaries of Product Characteristics. Intern. Med. J..

[31] D’Angio R.G. (1987). Nonsteroidal antiinflammatory drug-induced renal dysfunction related to inhibition of renal prostaglandins. Drug Intell. Clin. Pharm..

[32] Briguori C., Tavano D., Colombo A. (2003). Contrast agent—Associated nephrotoxicity. Prog. Cardiovasc. Dis..

[33] Choudhury D., Ahmed Z. (2006). Drug-associated renal dysfunction and injury. Nat. Clin. Pract. Nephrol..

[34] Markowitz G.S., Perazella M.A. (2005). Drug-induced renal failure: A focus on tubulointerstitial disease. Clin. Chim. Acta.

[35] Pannu N., Nadim M.K. (2008). An overview of drug-induced acute kidney injury. Crit. Care Med..

[36] Rossert J. (2001). Drug-induced acute interstitial nephritis. Kidney Int..

[37] St Peter W.L., Clark J.L., Levos O.M. (1998). Drug therapy in haemodialysis patients. Special considerations in the elderly. Drugs Aging.

[38] Jick H. (1977). Adverse drug effects in relation to renal function. Am. J. Med..

[39] Martin-Facklam M., Rengelshausen J., Tayrouz Y., Ketabi-Kiyanvash N., Lindenmaier H., Schneider V., Bergk V., Haefeli W.E. (2005). Dose individualisation in patients with renal insufficiency: Does drug labelling support optimal management?. Eur. J. Clin. Pharmacol..

